# Intergroup trust as a mediator between compassion and positive attitudes toward sexual minorities

**DOI:** 10.3389/fpsyg.2022.1015595

**Published:** 2022-12-05

**Authors:** Nikoleta Kuglerová, Miroslav Popper, Xenia Daniela Poslon

**Affiliations:** ^1^Faculty of Social and Economical Sciences, Institute of Applied Psychology, Comenius University, Bratislava, Slovakia; ^2^Institute for Research in Social Communication, Slovak Academy of Sciences, Bratislava, Slovakia

**Keywords:** compassion, self-compassion, intergroup trust, sexual minorities, attitudes

## Abstract

Nurturing compassion is not only beneficial for one’s well-being in terms of feelings and cognitions directed toward oneself, but it can also have positive effects on attitudes toward other people through associated humanity and recognition of the universality of suffering. Having compassion toward others may be particularly beneficial in intergroup relations, as minority and stigmatized groups often experience a lack of compassion from the majority. The present study (*N* = 244) examines the relation between self-compassion, compassion toward others, and the level of trust and positive attitudes toward members of sexual minorities. The results of path analysis suggest that the relationship between compassion for others and attitudes toward people belonging to sexual minorities is mediated by intergroup trust. Fostering compassion could therefore play an important role in increasing trust and improving attitudes toward the people belonging to stigmatized minorities.

## Introduction

In the early October evening this year, a 19-year-old high school student targeted and attacked people sitting in front of a well-known LGBT bar in Bratislava, injuring one and murdering two young people. Slovak National Crime Agency has since classified the shootings as a terrorist crime motivated by hatred toward people belonging to sexual minorities (Maishman, 2022). This hate crime demonstrates the prevalence of prejudice toward people belonging to sexual minorities, that is not only alarming in Slovakia, but also in other European countries. Based on 2019 Eurobarometer on discrimination (European Commission, 2019), the social acceptance of sexual minorities is still low in many European countries, with Slovak citizens scoring among the least accepting of LGBT people. According to a FRA survey (FRA-European Union Agency for Fundamental Rights, 2020), 77% of LGBT people in Slovakia refuse to hold their partner’s hand in public and only 26% of people identifying as LGBT openly disclose their sexual orientation or gender identity. This points to the severe lack of compassion toward people belonging to sexual minorities, that has many consequences not only when it comes to basic human rights, open hostility and discrimination, but also in the form of sexual minorities’ mental health, well-being, and feelings of security (Meyer and Frost, 2013).

Social psychologists are continuously looking for new ways to improve the position and well-being of stigmatized groups, while research is often focused on reducing the negative aspects of intergroup relations, such as majority members’ hostile feelings and prejudice toward various minority groups. Yet, Gonzalez et al. (2015) argue that including a more specific development of positive feelings and attitudes toward stigmatized minorities is also needed for promoting more positive intergroup relations, and especially prosocial and supportive behavior. So far, little attention has been paid to compassion, which could play a significant role in this process. The numerous benefits of compassion are not only in its role in caring for others (Kirby et al., 2017) and improving emotional intelligence (Barnard and Curry, 2011), but also in its wider impact on society through the associated humanity, prosocial behavior (Leiberg et al., 2011), and altruism (Neff and Pommier, 2013). There is now an increasing interest in studying the effects of interventions based on compassion toward others on outgroup attitudes and prosocial behavior (e.g., Hunsinger et al., 2014; Lueke and Gibson, 2016; Sinclair et al., 2016; Berger et al., 2018). However, we still lack understanding of what could promote feelings of compassion and what are the underlying mechanisms between compassion and attitudes toward others.

In the present study, we explore the possible associations between self-compassion, compassion for others, and attitudes toward sexual minorities. Being compassionate toward oneself could help promote feelings of compassion and concern for others, since a common mechanism in both compassion and self-compassion is the awareness that failure and suffering are part of human nature and all people are worthy of love and understanding (Neff, 2003). Nurturing self-compassion has been associated with greater compassion for humanity, concern for the suffering of others, as well as altruism and forgiveness (Neff and Pommier, 2013). Even though the research on compassion toward sexual minorities is scarce, there is reason to believe that both compassion toward others and oneself could be positively associated with attitudes toward suffering groups, including sexual minorities. Given that intergroup trust is essential for a positive perception of members of another group and positive intergroup relations (Halabi et al., 2021), and higher compassion may be associated with increased trust toward others, we also examined the role of intergroup trust in the relationship between compassion and attitudes toward sexual minorities.

### Compassion and self-compassion

Compassion is typically defined through its various attributes, mainly emotions, motivation, and disposition. Gilbert et al. (2017) emphasize that compassion is related to sensitivity regarding motivation and behavior, while Eisenberg and Spinrad (2004) associate compassion with a higher level of self-regulation and see it as a dispositional characteristic. Compassion can, therefore, be regarded as a multidimensional construct comprising cognitive, emotional, and behavioral components (Jazaieri et al., 2013; Strauss et al., 2016). Strauss et al. (2016) provide a more detailed description of each component and introduce five characteristics: (1) recognition of suffering and (2) understanding its universality (the cognitive component), (3) empathy for the person suffering in relation to emotional resonance and (4) tolerance of uncomfortable feelings evoked by witnessing suffering (the emotional component), and (5) motivation for taking action to alleviate suffering (the behavioral component).

Self-compassion is compassion turned inwards that we give ourselves during difficult life situations or when confronting our own failures and mistakes (Germer and Neff, 2013), and it could be considered as an extension of compassion toward the self, serving as an emotion regulation strategy (Neff, 2003). In describing self-compassion, Neff (2003) distinguishes three personality traits: (1) self-kindness (kind behavior toward oneself in case of pain or failure), (2) common humanity (perception that other people’s experiences are part of the larger human experience) and (3) mindfulness (the ability to hold painful thoughts and give them adequate attention). The definition of compassion toward others by Strauss et al. (2016) on the basis of the five above-mentioned components can also be applied to define self-compassion, since the recognition of suffering and understanding of its universality, empathy, the ability to tolerate unpleasant emotions and the motivation to do something to alleviate suffering can be directed toward oneself as well as toward others (Gu et al., 2017, 2020). Both compassion and self-compassion are considered to comprise the components of common humanity (Neff, 2003; Feldman and Kuyken, 2011) and mindfulness (Neff, 2003; Gilbert and Procter, 2006). Therefore, even though self-compassion is typically studied in terms of intrapersonal benefits, there may be similar mechanisms underlying compassion oriented toward others and self-compassion, and it is worth exploring the interpersonal or intergroup outcomes of higher self-compassion, as well as and its role in cultivating compassion in general.

Since self-compassion makes us realize that we all suffer, we are able to connect ourselves with others (Germer and Neff, 2013), and higher levels of self-compassion could be associated with increased compassion for others. Neff and Pommier (2013) point out that self-compassion is significantly correlated with other-focused concern (including compassion for humanity) and both compassion and self-compassion seem to be important for the development of emotional intelligence (Di Fabio and Saklofske, 2021). In fact, people higher in self-compassion are able to calm themselves in difficult situations without getting carried away by negative reactions (Neff, 2003) thus be more prepared to cope with other people’s suffering. Such emotional resilience and emotional regulation skills may lead to increased and healthier mechanisms of compassion toward others (Neff and Pommier, 2013), which could also help prevent compassion burnout (Heffernan et al., 2010). As Welp and Brown (2014) suggest, if we know how to be compassionate toward ourselves, we may be more equipped to be considerate and compassionate toward others as well.

### (Self-)compassion and attitudes toward outgroups

Even though empathy is more often at the forefront of research in intergroup relations (Stephan and Finlay, 1999), it is worth examining the social benefits of compassion for others (Seppala et al., 2013) that may also extend to those outside one’s ingroups. Compassion is often lower for outgroups, especially when one has higher ingroup preference. For example, compassion toward vulnerable groups, such as sexual minorities, was related to ingroup preference and identification or acquaintance with someone from the outgroup (Floyd et al., 2022). Sinclair et al. (2016) showed that compassionate love (defined as feelings, reactions, and behaviors aimed at expressing care and understanding of others) relates to anti-immigrant prejudice, while the relationship was mediated by inclusion of outgroup members in the self. Yet nurturing self-compassion, even though typically associated with improving one’s own well-being, can also increase the awareness of the common humanity, which may relate to compassion in general and more positive attitudes toward other people (Neff and Pommier, 2013). From a theoretical point of view, self-compassion is considered to favor openness and a positive orientation toward others for at least two reasons (Fuochi et al., 2018). Firstly, self-compassion does not imply self-centeredness (Neff et al., 2007) and its focus on compassionate feelings, caring attitude, and non-judgmental understanding, although directed to the self, might also foster compassion, acceptance, and openness toward others (Hoffmann et al., 2011; Neff and Pommier, 2013). Secondly, self-compassionate people experience failures, weaknesses, and sufferings as part of human nature, and thus perceive all humans (including the self) as worthy of compassion. Self-compassion may eliminate the boundaries between self and others, which generates a sense of connection (Neff, 2003; Neff and Seppälä, 2016) and increase sense of community (Akin and Akin, 2017). Self-compassion may also increase willingness to help others in need (Welp and Brown, 2014) and it was found to predict prosocial behavior (Yang et al., 2021). Furthermore, Yang et al. (2019) showed that tendency to trust others mediated the relationship between self-compassion and prosocial behavior in adolescents, and suggest that future studies should pay attention to other possible mediators, such as compassion for others.

Therefore, even though there is reason to believe other-oriented benefits of self-compassion could extend to more positive attitudes toward outgroups as well, studies that examine self-compassion in the context of intergroup relations are rare. For example, Verhaeghen and Aikman (2020) showed that self-compassion is positively related to the motivation to control prejudiced reactions and negatively to explicit prejudice, and based on their results, the authors suggest that compassion for others may be the mediating mechanism between these variables. Fuochi et al. (2018) explored different components of self-compassion and found that the common humanity component relates to empathic concern and attitudes toward a person in need belonging to a stigmatized group, while mindfulness was related to a reduction in personal distress when witnessing others suffering. Self-compassion therefore might relate to more positive attitudes toward stigmatized groups based on the increased awareness of common humanity, which may activate the perception of shared or superordinate identity, known to have positive effects in intergroup context (Gaertner et al., 1999).

Other research in this domain focused on practices and interventions aimed at increasing different aspects of compassion in general and/or self-compassion. Cultivating mindfulness and compassion may increase the ability to recognize suffering, whether of self or others (Gilbert and Procter, 2006). For example, Berger et al. (2018) succeeded in reducing Israeli-Jewish pupils’ prejudice toward the Israeli-Palestinian outgroup with an intervention aimed at cultivating mindfulness and compassion. Loving-kindness meditation, that cultivates both compassionate and self-compassionate behavior, was found to reduce intergroup anxiety, raise interest in future contact, and lead to more positive explicit attitudes toward the homeless (Parks et al., 2014) as well as improve implicit attitudes toward different stigmatized groups (Kang et al., 2014, 2015). Hunsinger et al. (2014) found that people who actively practice compassion-centered meditation, characterized by the cultivation of a positive emotional state toward others, displayed lower levels of racial prejudice compared to those who had no experience with meditation. The above research findings offer support for the assumption that fostering greater compassion oriented toward the self or the others can improve attitudes toward various stigmatized groups. However, little is known about the mechanisms underlying these effects, and more research is needed to distinguish between the effects of compassion for others and compassion for oneself on outgroup attitudes.

### Intergroup trust as an underlying mechanism between compassion and attitudes toward outgroups

Until now, little attention has been paid to the psychological mechanisms through which compassion might relate to intergroup attitudes. For example, Liu and Wang (2010) found that the relationship between compassion and cooperative goals is mediated by participants’ trust. Compassionate goals, which focus on supporting others, also predict an increase in interpersonal trust, and at the same time, people with such goals have increased self-compassion (Crocker and Canevello, 2008). People who enjoy interacting with others tend to perceive them as trustworthy and nonthreatening (Seppala et al., 2013) and there is also a strong positive relationship between perception of other people’s qualities and trust (Christie et al., 2015). Jones (2019) even proposes the concept of  ‘compassionate trust’ which she understands as a “hopeful trust, driven by compassion for others.” Although there is little research on the direct connection between compassion and trust, based on the above-mentioned studies, we can assume that both trust and compassion are needed for one to effectively manage vulnerability.

Trust is therefore offered as a possible candidate for an underlying mediator between compassion and attitudes toward minority or stigmatized groups. Since intergroup relations are often characterized by mutual distrust and contempt (Batson and Ahmad, 2009), mutual compassion between members of different groups is difficult to achieve. People tend to trust others if they share category memberships, i.e., trust is greater toward the ingroup than outgroup members (Yuki et al., 2005). On the other hand, achieving trust toward people belonging to the outgroup can promote more positive intergroup relations (Halabi et al., 2021) as trust belongs to the most important psychological conditions for developing positive relationships between groups (Tropp, 2008). Ekici and Yucel (2015) showed that trust is negatively correlated with prejudice and increasing trust thus may be associated with more positive intergroup attitudes. Grütter et al. (2018) found that changes in intergroup trust and sympathy predict attitudes toward the outgroup, while Turner et al. (2007) similarly showed that outgroup attitudes can be improved by increasing empathy and intergroup trust. Moreover, outgroup trust is a strong predictor of behavioral tendencies toward the outgroup (Tam et al., 2009).

### The present study

So far, there has been a lack of research examining the relationship between compassion oriented toward the self and the others and outgroup attitudes, and the psychological mechanisms that could explain this relationship. Moreover, the scarce research in this domain has mostly been focused on attitudes toward the homeless or ethnic outgroups. In an effort to fill this gap, the aim of the present study is to test a path model with the assumption that self-compassion may relate to compassion toward others and positively predict attitudes toward people belonging to sexual minorities (lesbian, gay, and bisexual people) through intergroup trust as a mediator. Given that men often report more negative attitudes toward sexual minorities than women, we also controlled for participants’ gender (Ratcliff et al., 2006; Parrott, 2009).

## Methods

### Research sample

Our initial sample consisted of *N* = 323 participants. However, as our objective was to examine the majority member’s attitudes toward people belonging to sexual minorities, we removed participants that did not identify as heterosexual (*N* = 79).^1^ The final sample consisted of *N* = 244 participants, aged between 18 and 53 years (*Mean* = 25.6; *SD* = 5.99; 78.7% women). Based on the number of predictors, this sample size is sufficient for 0.80 power to detect a medium effect size in a multiple regression framework, given the number of predictors (Cohen, 1992). Participants were recruited on social networks, using the convenience sampling method. Most participants were of Slovak ethnicity 91.8% (*N* = 224; the other 10% were of Czech and Hungarian ethnicity). In terms of educational attainment, 70.9% (*N* = 173) had a university degree, 28.3% (*N* = 69) had completed secondary education, and two participants were still attending secondary education. The study was reviewed and approved by the Ethical Committee of the Institute for Research in Social Communication of the Slovak Academy of Sciences.

### Measures

We used the Slovak version (Halamová and Kanovský, 2021) of the self-report Sussex-Oxford Compassion for Others Scale (Gu et al., 2020) to measure levels of compassion for others, and the corresponding Slovak version (Halamová and Kanovský, 2021) of the Sussex-Oxford Self-compassion Scale (Gu et al., 2020) to measure levels of self-compassion. The original scales were developed as comprising five dimensions: (1) recognizing suffering, (2) understanding the universality of suffering, (3) feeling for the person suffering, (4) tolerating uncomfortable feelings, and (5) acting or being motivated to act to alleviate suffering. Each scale contains 20 statements in total, such as “I recognize when other people are feeling distressed without them having to tell me” (Compassion for Others Scale) or “I’m good at recognizing when I’m feeling distressed” (Self-compassion Scale). However, Halamová and Kanovský (2021) explored the factor structure of the scales on a Slovak sample and found that in the case of Self-compassion Scale, there were two overarching factors over the original five factors: rational compassion (containing recognizing suffering and understanding the universality of suffering) and emotional/behavioral compassion (containing feeling for the person suffering, tolerating uncomfortable feelings, and acting or being motivated to act to alleviate suffering). When it comes to Compassion for Others Scale, the authors demonstrated essential unidimensionality, and suggest that a total score (one-factor model) can be safely used. The reliability of the two factors in the case of Self-compassion Scale and the total score for the Compassion for Others Scale was good to acceptable in our sample: ω = 0.753 for rational self-compassion; ω = 0.866 for emotional/behavioral self-compassion and ω = 0.907 for compassion for others. Respondents indicated their agreement with the statements on a five-point Likert scale (1 = not at all true, 5 = always true). Higher values indicate higher (self-)compassion.

To measure attitudes toward people belonging to sexual minorities, we used the feeling thermometer (Esses et al., 1993). Participants were asked to describe their own feelings toward people belonging to sexual minorities on a scale from 0 (denoting cold, negative feelings) to 100 (warm, positive feelings). Finally, we adapted the scale measuring intergroup trust from the INTERMIN questionnaire (Lášticová and Findor, 2016), originally developed in Slovakia to measure prejudice toward the Roma. Participants rated their agreement with two statements: “Most homosexual or bisexual people are trustworthy” and “I generally trust homosexual or bisexual people” (ω = 0.855) on a 7-point Likert scale (1 = completely disagree, 7 = completely agree). Higher values indicate more trust toward people belonging to sexual minorities.

We additionally measured socio-demographic variables for descriptive purposes, such as age, education, ethnicity, sex, gender identity, and sexual attraction.

### Data analysis

Path analysis was conducted using Mplus version 8.7 (Muthén and Muthén, 1998). Goodness of fit of the models (Hu and Bentler, 1999) was assessed using the following indexes: the root mean square error of approximation (RMSEA < 0.08), the CFI (Comparative Fit Index > 0.90), the TLI (Tucker-Lewis Index > 0.90), and the standardized root mean square residual (SRMR < 0.08).

## Results

Descriptive statistics and correlations are shown in Table 1. Bivariate correlations indicate that both rational self-compassion and emotional/behavioral self-compassion are positively associated with compassion for others, supporting our assumptions. However, contrary to our expectations, there were no associations between the two self-compassion factors and trust or attitudes toward people belonging to sexual minorities. Only participants’ self-reported compassion for others was positively associated with the levels of trust and attitudes toward sexual minorities. Finally, as expected, intergroup trust was positively associated with attitudes toward people belonging to sexual minorities. Participants’ gender was positively associated with both trust and attitudes toward sexual minorities, as women tended to report higher trust and more positive attitudes.

**Table 1:** Descriptive statistics and correlations between the study variables.

	Mean	SD	1	2	3	4	5	6
1. Rational self-compassion	4.47	0.42	-					
2. Emotional/behavioral self-compassion	3.20	0.69	**0.33** ^***^	-				
3. Compassion for others	4.06	0.50	**0.50** ^***^	**0.14** ^*^	-			
4. Trust toward sexual minorities	5.19	1.57	0.03	0.06	**0.21** ^***^	-		
5. Attitudes toward sexual minorities	63.3	30.8	−0.02	0.03	**0.20** ^**^	**0.54** ^***^	-	
6. Gender	-	-	−0.03	**−0.14** ^*^	0.11	**0.25** ^***^	**0.27** ^***^	-

Gender: 0 = male, 1 = female.

* *p < 0.05;*

** *p < 0.01;*

*** *p < 0.001*. Bolded values are significant at *p* < 0.05.

**Figure 1:**
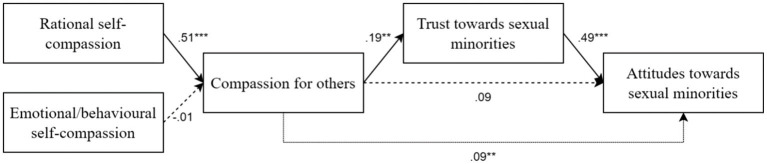
Path model with standardized path coefficients. For purposes of readability, error variances are omitted. Dashed lines represent non-significant paths, dotted lines represent indirect effects. **p < 0.01, ***p < 0.001.

We estimated a path model using maximum likelihood estimation and manifest variables where two components of self-compassion predict compassion for others, and compassion for others predicts attitudes toward sexual minorities as outcome variable, while controlling for participants’ gender.^2^ Trust toward sexual minorities was entered as a mediator between compassion for others and attitudes toward sexual minorities. Since neither rational nor emotional self-compassion correlated with trust or attitudes toward sexual minorities, we did not estimate any direct or indirect paths from self-compassion factors to attitudes toward sexual minorities.^3^ The model was bootstrapped with 5,000 resamples to obtain 95% confidence intervals. The model had a good fit to the data (*χ2* = 11.5, *DF* = 6, *p* < 0.074, *CFI* = 0.97, *TLI* = 0.94, *RMSEA* = 0.06, *SRMR* = 0.04). Figure 1 presents the standardized path coefficients of the model.

The results showed that compassion for others was positively and significantly predicted by rational self-compassion (*β* = 0.51, *SE* = 0.05, *p* < 0.001), but not by emotional/behavioral self-compassion (*β* = −0.01, *SE* = 0.06, *p* = 0.904). The direct path from compassion for others to attitudes toward sexual minorities in the model was not significant (*β* = 0.09, *SE* = 0.06, *p* = 0.147). However, the results of mediation analysis showed that compassion for others significantly predicted trust toward sexual minorities (*β* = 0.19, *SE* = 0.06, *p* = 0.002), which in turn predicted attitudes (*β* = 0.49, *SE* = 0.06, *p* < 0.001), with a significant indirect effect (*β* = 0.09, *SE* = 0.03, 95% CI [0.03, 0.06]), indicating full mediation. In terms of the control variable, gender significantly predicted both trust toward sexual minorities (*β* = −0.23, *SE* = 0.06, *p* < 0.001) and attitudes toward sexual minorities (*β* = −0.13, *SE* = 0.06, *p* = 0.018), with female participants reporting more intergroup trust and more positive attitudes than male.

## Discussion

The aim of the present study was to examine the relationships between compassion oriented toward oneself and the others, intergroup trust, and attitudes toward sexual minorities. First of all, our findings suggest that there is a positive relationship between self-compassion and compassion toward others, and provide further support in justification to distinguish between two dominant factors in SOCS-S (Halamová and Kanovský, 2021), since based on our results, only the rational component of self-compassion positively and significantly predicted compassion toward others. This could imply that recognizing one’s own suffering and understanding the universality of suffering in human experience might be more transferable to another person than feeling for and connecting with one’s own distress, tolerating uncomfortable feelings, and acting to alleviate one’s own suffering. One of the possible explanations is that highly compassionate people need to regulate the level of their emotional load and therefore make more use of the cognitive component of compassion, primarily perspective taking, which predicts compassion satisfaction, but does not lead to compassion exhaustion (Duarte et al., 2016).

Consistent with relatively rare findings (Welker et al., 2014; Sinclair et al., 2016; Berger et al., 2018), our research suggests that there is a link between compassion and intergroup attitudes. However, contrary to our assumptions, neither of the two self-compassion components were associated with trust or attitudes toward sexual minorities. Even though rational self-compassion was positively associated with compassion for others in general, our results suggest that there is no unique contribution of self-compassion to outgroup attitudes. Therefore, only compassion oriented toward others seems to be connected to more positive attitudes toward minority and stigmatized groups, and it may not be surprising that one can have positive attitudes toward outgroups without cultivating self-compassion. Yet, one of the main reasons to cultivate both compassion and self-compassion is that it may prevent compassion burnout (Heffernan et al., 2010), and people high in self-compassion may be more equipped to help those in need. For this reason, it may be fruitful to examine the specific effects of high compassion toward others on one’s well-being, which is typically conducted in the domain of helping professions (Heffernan et al., 2010), also in the context of intergroup relations.

Our results further indicate that intergroup trust plays a mediating role between compassion toward others and attitudes toward people belonging to sexual minorities. This supports the assumption that trust belongs to one of the most important psychological conditions for developing positive relationships between groups (Yuki et al., 2005; Tropp, 2008), can strengthen cooperation (Liu and Wang, 2010), and interaction with others (Seppala et al., 2013) and promote more positive intergroup relations (Turner et al., 2007; Ekici and Yucel, 2015; Zarins and Konrath, 2017; Grütter et al., 2018; Halabi et al., 2021). Our results are also in line with Crocker and Canevello (2008) who found that individuals who scored high in self-compassion have more compassionate goals, encourage interpersonal trust with others and provide them with greater social support.

Based on findings from initial experimental studies, and the correlational data of our study, future research should explore the effectiveness of compassion and mindfulness interventions, not only for alleviating one’s own suffering (Gilbert et al., 2017), but also for improving attitudes toward others, including various stigmatized minorities. Future studies may also specifically explore the assumed effect of increased awareness of the common humanity in the context of intergroup relations, as there is reason to believe it may relate to perceptions of shared identity with the outgroup.

### Limitations

Although the research sample spanned a wide age range, it was not representative of various demographic characteristics. Further research on representative samples and in countries with different normative climate is needed to support our findings. Furthermore, the scales used in our research were self-report and measured explicit intergroup attitudes and it would be useful to conduct additional research using implicit prejudice indicators or measures of modern prejudice. Finally, our study is correlational, and future research should utilize experimental designs and priming or training compassion in order to establish the causal relationships between compassion, trust and intergroup attitudes toward minority and stigmatized groups.

## Data availability statement

The raw data supporting the conclusions of this article will be made available by the authors, without undue reservation.

## Ethics statement

The studies involving human participants were reviewed and approved by Ethical Committee of the Institute for Research in Social Communication, Slovak Academy of Sciences, Bratislava, Slovakia. The patients/participants provided their written informed consent to participate in this study.

## Author contributions

MP proposed the conception of the study. NK and MP designed the study. NK and XP analyzed the data. NK drafted the first version of the paper. MP and XP participated in the writing of the posterior versions of the manuscript. All authors contributed to the article equally and approved the submitted version.

## Funding

The research was conducted with the support of VEGA Grant 1/0075/19: “The Intercultural Aspects of Compassion, Self-Compassion, and Self-Criticism and the Testing of Their Influence” and VEGA Grant 2/0035/21 “Family Constellations Involving Biological and Non-biological Children”.

## Conflict of interest

The authors declare that the research was conducted in the absence of any commercial or financial relationships that could be construed as a potential conflict of interest.

## Publisher’s note

All claims expressed in this article are solely those of the authors and do not necessarily represent those of their affiliated organizations, or those of the publisher, the editors and the reviewers. Any product that may be evaluated in this article, or claim that may be made by its manufacturer, is not guaranteed or endorsed by the publisher.

## Footnotes

^1^The initial sample was collected as part of a doctoral dissertation with the aim to explore the differences in self-compassion between heterosexual and non-heterosexual individuals.

^2^We checked the correlations between attitudes toward sexual minorities and participants’ age and education as potential control variables. Participants’ age did not correlate with any of the variables of interest. Education only weakly correlated with attitudes toward sexual minorities (r = 0.15, *p* = 0.018) and trust (r = 15, *p* = 0.016), and there was no significant correlation between education level and compassion toward others. Therefore, only gender was included as a control variable.

^3^We also tested a path model without compassion for others, estimating direct effects from the two self-compassion components to attitudes toward sexual minorities, mediated by trust. However, neither direct nor indirect paths from the two self-compassion components to attitudes toward sexual minorities were significant, indicating that, contrary to our expectations, self-compassion does not uniquely contribute to trust or attitudes toward sexual minorities, and seems to be only positively associated with higher compassion for others in general.
